# The Potential of *Rhizoctonia*-Like Fungi for the Biological Protection of Cereals against Fungal Pathogens

**DOI:** 10.3390/plants10020349

**Published:** 2021-02-12

**Authors:** Dominik Bleša, Pavel Matušinský, Romana Sedmíková, Milan Baláž

**Affiliations:** 1 Department of Plant Pathology, Agrotest Fyto, Ltd., 76701 Kroměříž, Czech Republic; matusinsky@vukrom.cz; 2 Department of Experimental Biology, Faculty of Science, Masaryk University, 62500 Brno, Czech Republic; 375890@mail.muni.cz (R.S.); balaz@sci.muni.cz (M.B.); 3 Department of Botany, Faculty of Science, Palacký University in Olomouc, 78371 Olomouc, Czech Republic

**Keywords:** *Rhizoctonia*-like fungi, biocontrol, *Fusarium culmorum*, *Serendipita indica*, *Microdochium bolleyi*, *Ceratobasidium* sp., *Tulasnella* sp., endophyte

## Abstract

The use of biological control is becoming a common practice in plant production. One overlooked group of organisms potentially suitable for biological control are *Rhizoctonia*-like (*Rh-*like) fungi. Some of them are capable of forming endophytic associations with a large group of higher plants as well as mycorrhizal symbioses. Various benefits of endophytic associations were proved, including amelioration of devastating effects of pathogens such as *Fusarium culmorum*. The advantage of *Rh*-like endophytes over strictly biotrophic mycorrhizal organisms is the possibility of their cultivation on organic substrates, which makes their use more suitable for production. We focused on abilities of five *Rh*-like fungi isolated from orchid mycorrhizas, endophytic fungi *Serendipita indica*, *Microdochium bolleyi* and pathogenic *Ceratobasidium cereale* to inhibit the growth of pathogenic *F. culmorum* or *Pyrenophora teres* in vitro. We also analysed their suppressive effect on wheat infection by *F. culmorum* in a growth chamber, as well as an effect on barley under field conditions. Some of the *Rh*-like fungi affected the growth of plant pathogens in vitro, then the interaction with plants was tested. Beneficial effect was especially noted in the pot experiments, where wheat plants were negatively influenced by *F. culmorum*. Inoculation with *S. indica* caused higher dry shoot biomass in comparison to plants treated with fungicide. Prospective for future work are the effects of these endophytes on plant signalling pathways, factors affecting the level of colonization and surviving of infectious particles.

## 1. Introduction

Endophytic capabilities of fungi forming orchid mycorrhizas were observed many times in different plant families [1,2,3]. The majority of these orchid symbionts belongs into a wide group of fungi mostly from phylum Basidiomycota, few members being from Ascomycota [4]. In Basidiomycota phylum, we can distinguish one paraphyletic subgroup generally called *Rhizoctonia*-like (*Rh*-like) fungi or “rhizoctonias”, which include members of the families Ceratobasidiaceae and Tulasnellaceae and order Sebacinales. The morphological definition of *Rh*-like fungi includes monilioid cells, unimpaired parenthosomes getting into doliporus septa and right-angle branching of hyphae [4,5,6]. The former methods of their identification were based mainly on morphological parameters, while recent identification of *Rh*-like fungi is based on the molecular methodology as the sequencing of, e.g., ITS regions of rDNA [7]. Genus *Rhizoctonia* was established historically by de Candolle (1815) and is coherent to *Rhizoctonia solani*, now this genus is justified as a paraphyletic group encompassing species from different clades. Their pathogenicity depends on the genotype of a fungal strain and host plant species [8]. The fungi from *Rh*-like group are cosmopolitans mostly with saprotrophic abilities [4], but the life strategy and other ecological aspects of these endophytes are not well-known [3].

Most of the research was done on the members of the order Sebacinales, especially on the genus *Serendipita*. The effects and mutualistic ability (growth promotion) was observed mostly in *Serendipita indica* (syn.: *Piriformospora indica*), *Serendipita herbamans*, and *Serendipita vermifera* (syn.: *Sebacina vermifera*) [2,7,9,10,11]. These species also point to the potential in applied research or agriculture due to low host specificity [3,12,13,14]. The order Sebacinales is known to be involved in various mycorrhizal symbioses and endophytic associations. However, other fungi forming orchid mycorrhiza are also capable of initiation endophytic symbiosis [3,7,15]. As the ability of many *Rh*-like fungi to colonize roots of different plant species differ, *Rh*-like fungi do affect the species structure of vegetation [16]. The substrate competition and niche occupation may play a role in spreading of pathogen or disease outbreaking, but the disease protective effects of *Rh*-like fungi may also encompass systemic resistance or systemically acquired resistance [12,17]. Isolates of *Rh*-like fungi forming a mycorrhizal symbiosis with orchids are used due to their non-pathogenic action for biological protection of plants against their phylogenetically related representatives, which cause significant economic losses [18,19]. Orchid tissues contain a wide range of fungi that grow endophytically, which have protective effects against plant pathogens [20].

With the current emphasis on the sustainability of food production and the reduction of the use of chemical plant protection products, the study of the properties of endophytic organisms in the context of biological protection and the ability to improve crops is a key issue. Biological plant protection preparations offer a variety of effects, but it presupposes knowledge of broader context at the level of the plant and of the whole community [17,21]. Usually, the effect of chemical protection is faster, more effective and in many cases the use of specific chemical substances is inevitable [22,23]. Chemical protection can also affect non-target organisms, the quality of life of the surrounding population, or cause a phytotoxic effect on plants [24]. In contrast, biological protection through *Rh*-like fungi can mediate the translocation of a nutrient pool or provide other beneficial features [15,25,26,27]. If biological control agents are to be used on a wider scale in agriculture, they should be able to reduce the incidence of major cereal pathogens such as *Fusarium* species complex.

*Fusarium* species, especially *F. culmorum*, are a group of fungal plant pathogens that are responsible for a decrease of yield and quality of grain production constantly every year with rarely epidemic duration [28]. *F. culmorum* causes two diseases on wheat: Fusarium root rot and Fusarium head blight. Fusarium root rot symptoms are pre- and post-emergence seedling death, or brown discolouration on the coleoptiles, roots and the pseudostem [28]. Fusarium head blight symptoms include partial head blighting, with the prematurely bleached spikelets, or blighting of the entire head [28,29]. *F. culmorum* is pathogen causing Fusarium head blight in most places in Europe and is considered to be one of the main pathogens of wheat worldwide [30], which significantly reduces the yield and quality of harvested wheat and barley grains [28]. Lower yield production is mainly caused by *Fusarium*-damaged kernels or spikelet sterility [31,32,33]. The quality of contaminated grains is also lower due to the reduced content of nutrients and due to the presence of mycotoxins, which are toxic for humans and animals. The costs of fungicides or treatment of affected animals are enormous. Additionally, the reduction in biomass and grain yield noticeably decreases income in the agriculture industry [34,35,36]. The manifestations of the disease are influenced by many factors such as temperature, humidity, genetic constitution both of the host and the pathogen, biotic or abiotic stresses, preceding crop, rotation of crops or agrotechnical management [17,37,38,39]. These environmental factors play an important role in the management of the disease by fungicides or by biocontrol agents [38,40].

The world’s most serious pathogens can also include pathogenic representatives of *Rh*-like fungi. *C. cereale* as a teleomorph of *R. cerealis* causing a sharp eye spot on the bases of cereals, especially wheat. Severe infection can result in the death of young tillers or cause lodging of stems [41]. Infestation with this pathogen allows other fungi to colonize host tissues, include highly virulent pathogens represented by *F. culmorum*, or other weaker or non-pathogenic species of fungi such as *Microdochium bolleyi* [41]. *M. bolleyi* can be considered as a potential biocontrol agent against aggressive soil-borne pathogens in cereal crops [42].

The main objective of this work is to observe the effects of fungi forming a mycorrhizal symbiosis with orchids against cereal pathogens. In this study, three independent experiments were carried out. We analysed the competitive abilities of varia *Rh*-like fungi, specifically five isolates of orchid mycorrhizal fungi from the genera *Tulasnella* and *Ceratobasidium*, endophytic *M. bolleyi,* and pathogenic *C. cereale* on the growth of pathogenic fungi *F. culmorum* and *Pyrenophora teres* under in vitro conditions. We also tested the abilities of the same *Rh*-like fungi and a well-known beneficial endophyte *S. indica* to ameliorate Fusarium root rot of wheat caused by *F. culmorum* grown in a growth chamber and the introduction of these fungi into field conditions for qualitative and quantitative yield parameters and the occurrence of barley diseases. The effects of these microorganisms were compared with fungicidal treatment with standard seed dressing fungicide containing prochloraz and triticonazole (Kinto Duo).

## 2. Results

### 2.1. Competitive In Vitro Test

**Figure 1:**
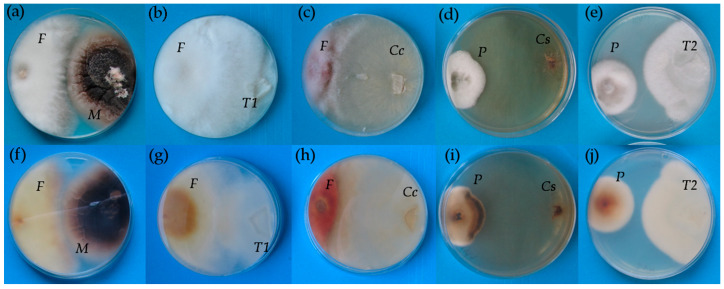
The growth of co-occurring *Microdochium bolleyi* and *Fusarium culmorum* (**a**,**f**), *Tulasnella* sp. 2016/11 and *F. culmorum* (**b**,**g**), *Ceratobasisium cereale* and *F. culmorum* (**c**,**h**), *Ceratobasidium* sp. 2015/1 isolate and *Pyrenophora teres* (**d**,**i**) and *Tulasnella* sp. 2015/2 and *F. culmorum* (**e**,**j**). Top view (**a**–**e**) and bottom view (**f**–**j**) on the same Petri dishes with potato-dextrose agar. *F*—*F. culmorum*, *M*—*M. bolleyi*, *T1*—*Tulasnella* sp. 2016/11, *Cc*—*C. cereale*, *P*—*P. teres*, *Cs*—*Ceratobasidium* sp. 2015/1, *T2*—*Tulasnella* 2015/2.

All endophytic fungi except *M. bolleyi* did not form an inhibition zone at the site of contact with the pathogenic *F. culmorum* (Table 1). The growth of the same pathogen was decreased only by the presence of *C. cereale*, which also caused the colour change of *F*. *culmorum* mycelium into the red (Figure 1**c**,**h**). The degradation of *P. teres* colonies manifested as darkening of the mycelium was initiated by the presence of three *Rh*-like fungi, *Ceratobasidium* sp. isolate 2015/1, *C. cereale*, and *Tulasnella* sp. isolate 2016/11.

### 2.2. Pot Experiments

#### 2.2.1. Experiment 1

**Figure 2:**
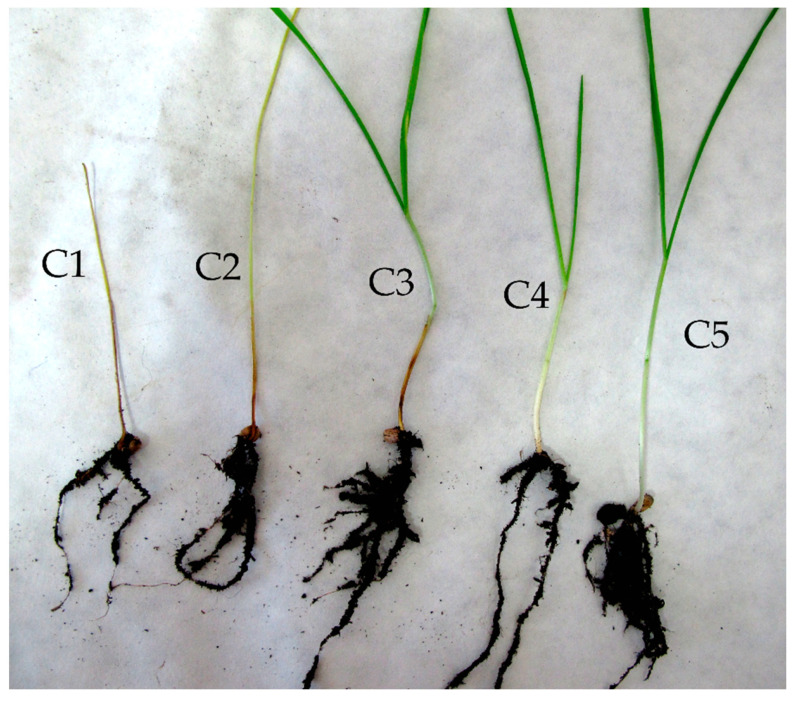
Five categories of plants depending on the degree of damage caused by the pathogen *Fusarium culmorum.* Categories 1 and 2 include necrotic (C1) and chlorotic (C2) germinated plants. Category 3 marks plants with symptoms like the browning of root neck (C3). Category 4—plants with a little brown spot on the top of the root neck (C4). Category 5 indicates plants with no visual symptoms of pathogen presence (C5). Categories 1–5—all plants emerged, categories 3–5—vital plants, category 5—symptomless plants.

There were highly statistically significant (*F* = 35.084; *p* < 0.001) differences in the number of plants emerged in different treatments. The highest numbers (89.3 ± 2.1, *n* = 3) were achieved for grains treated with the fungicide Kinto Duo, this value being significantly different from mean values of all other treatments. Similar pattern was found for the numbers of vital plants (*F* = 56.130; *p* < 0.001). Almost all plants (89.0 ± 2.0, *n* = 3) germinated from fungicide treated grains were vital, i.e., lacking any symptoms of shoot necrosis or chlorosis caused by *F. culmorum* (Figure 2). Wheat plants inoculated with different endophytes showed statistically significant differences in the colonization of their roots (*F* = 3.524; *p* = 0.034). The results are shown in Table 2. The highest colonization was achieved for plants inoculated with *Tulasnella* 2016/2, 12.3 ± 4.9% (*n* = 3) of root length (Table 3).

#### 2.2.2. Experiment 2

Neither any *Rh*-like fungus nor the fungicide Kinto Duo has a statistically significant effect (*F* = 2.234; *p* = 0.075) on the number of plants emerged. The overall mean of emerged plants was 83.2 ± 7.2 (*n* = 27). The number of plants without any sign of infestation, i.e., symptomless plants (*F* = 8.262; *p* < 0.001) was highest for grains treated with the fungicide Kinto Duo (47.3 ± 22.7, *n* = 3). The number of vital plants, i.e., plants without necrotic and chlorotic changes in shoots (Figure 2) was significantly higher for Kinto Duo treatment (88.7± 3.1, *n* = 3) compared to all the other treatments. The impact of treatments on shoot dry mass in a pot was not significant (*F* = 1.712; *p* = 0.163), the overall mean value was 1.0 ± 0.2 g (*n* = 27). However, if the dry mass of shoots in a pot was divided by the number of emerged plants, there were statistically significant differences among treatments (*F* = 3.951; *p* = 0.007), the smallest value found for plants emerged from Kinto Duo treated grains. The results are shown in Table 4. There were also statistically significant differences in wheat plant roots colonization by different endophytes (*F* = 46.018; *p* < 0.001). The highest colonization rate was observed for *Tulasnella* 2016/11, which occupied 83.3 ± 4.2% (*n* = 3) of root length (Table 3).

#### 2.2.3. Experiment 3

There were highly statistically significant (*F* = 7.279; *p* < 0.001) differences in the number of plants emerged in different treatments. The highest numbers were achieved by the endophyte treatment *S. indica* (99.3 ± 1.5, *n* = 3), while the treatment with the pathogenic strain *C. cereale* reached the lowest values (56.7 ± 3.1, *n* = 3). The number of vital plants without necrotic and chlorotic changes in shoots was statistically significant (*F* = 28.528; *p* < 0.001) as the effect of the experimental treatment. The highest number of vital plants was observed in plants colonized by the fungus *S. indica* (97.3 ± 2.3, *n* = 3). The treatment with pathogenic strain *C. cereale* had the lowest number of vital plants (5.0 ± 3.6, *n* = 3). The impact of treatments on the shoot dry mass in a pot (*F* = 17.342; *p* < 0.001) and the shoot dry mass related to the number of plants (*F* = 3.845; *p* = 0.004) were statistically significant. The values of the significant parameters are in Table 5. Inoculated wheat plants showed a statistically significant difference in the root colonization (*F* = 14.956; *p* < 0.001). The highest colonization rate had treatment *Tulasnella* 2016/11 with 87.7 ± 3.8% (*n* = 3) of root length (Table 3).

### 2.3. Field Experiment

The shoot fresh mass per plant (*F* = 0.548, *p* = 0.796; mean value 41.6 ± 13.8 g, *n* = 96) and the dry shoot mass (*F* = 0.490, *p* = 0.840; mean value 11.9 ± 4.0 g, *n* = 96) did not differ statistically significantly between treatments. During the season, occurrence of pathogens causing crown rot remained unmanifested, but one month before harvest, wheat leaf rust (*Puccinia* spp.) appeared as the main pathogen. The manifestation of the disease or the degree of plant infestation was related to plant phase and disease occurrence in our region. The yield of grain (*F* = 0.275, *p* = 0.958; mean value 7.5 ± 1.1 t/ha, *n* = 32), and qualitative parameters as the bulk density (*F* = 0.313, *p* = 0.930; 53.6 ± 2.7 kg/hL, *n* = 16), the weight of a thousand grains, (*F* = 0.866, *p* = 0.569; 38.3 ± 3.9 g, *n* = 16) did not differ between the individual treatments. There were statistically significant differences in barley plant roots colonization by different endophytes (*F* = 9.151; *p* < 0.001). The highest colonization rate was observed for *Tulasnella* 2016/11, which occupied 29.3 ± 16.4% (*n* = 3) of root length (Table 3).

## 3. Discussion

This work is a part of the research on endophytic interactions of a group of fungi generally called *Rh*-like fungi. Representatives of this group can be found across continents in diverse ecosystems, where their functions remain unexplored [7] and are studied mainly in relation to plant-pathogen interactions [17] and relation to orchid mycorrhiza [27,43]. Testing of local species as potential biocontrol agents is becoming more and more important. Similar cosmopolitan organisms are arbuscular mycorrhizal fungi, which as a natural part of ecosystems represent a promising opportunity in sustainable agriculture and plant protection. However, their limitation is the difficulty of cultivation as they are obligatory biotrophs [44,45,46,47]. In contrast, the endophytic fungi used in the experiment as well as many of other *Rh*-like fungi can be cultivated on a large scale on an organic substrate, which makes their potential use more efficient and cheaper.

In vitro testing is commonly used as a first approach to assess traits of microorganisms studied [48,49]. Additionally, in our study, Petri dish tests were useful for comparison of *Rh*-like fungi, which were isolated from orchid roots as mycorrhizal fungi with two other *Rh*-like fungi as regard their abilities to compete against selected pathogens in competition. *C. cereale*, which is generally considered as a plant pathogen [50,51], was in the current study the only isolate able to compete with both the pathogenic fungi used, i.e., *F. culmorum* and *P. teres*, due to the action of metabolites, which uses as a pathogen to attack plants [52]. Only two of five isolates from orchid roots were able to cause darkening of the *P. teres* mycelia, which is a sign of degradation, but the reason for the action of only two isolates from this phylogenetically and ecologically related group of fungi remains unexplained and requires a more thorough analysis of metabolite interactions.

Although in vitro testing may indicate potentially efficient isolates of *Rh*-like fungi, which we were able to confirm against *F. culmorum* only in two isolates, in the case of *P. teres* in three, so this proves the effectiveness of the isolates only partially, and it is necessary to test them under more realistic growth systems, especially with the target plants species. We used the pot experiments as a level suitable before the field application of obtained novel *Rh*-like endophytes. Isolates whose effect against *F. culmorum* was evident in in vitro tests did not show any protective effect in the pot experiments, besides the treatment of *C. cereale* due to its pathogenic nature reduced the values of the evaluated parameters compared to the plants treated only with *F. culmorum*. Our results stay in accordance with observations of Lemańczyk and Kwaśna [41] who found that *C. cereale* can reduce the resistance of plants to other pathogens. Biological protection of plants through these endophytes does not only have to take place through direct competition with the pathogen, but also by the induction of plant defence mechanisms [17,53]. The mechanism of induction of the defence reaction of our isolates is unknown, but the signalling particles may be fragments of cell walls during the formation of an endophytic association [54].

The early stages of cereal development are well documented by the infectious pressure of fungi causing crown rot in the habitat [28,52]. The first and second pot experiments simulated the natural reproductive cycle of the *Fusarium* spp., in which the pathogen is present in the infected grains or its infectious particles are in the substrate [28]. According to the analyses described by Blanco et al. [55], the infected grain is the direct source of the earliest infections and allows the fungus to attack the sprouts. This is because the infectious particles are located under layers of the seed testa, which is a common way of transmitting of pathogens [55,56]. As we suspect, in our studies it enabled the pathogen easier enters the plant tissues compared to endophytes provided in both mentioned experiments from the outside. The third experiment simulated the infectious pressure of a pathogen, for which post-harvest crop residues serve as a source of spread, so the main infectious pressure comes at the seedling stage [57]. In this experiment, the infection could occur after the germination of plants and do not affect the early stages of an endophytic association. This delayed pathogen application did not affect the values of colonization despite our expectations, but it may have played a role in the plant development due to sudden stress compared to previous experiments, which led to a later harvest of Experiment 3 versus Experiment 2 to maintain the same growth stage of wheat. The colonization rate was reduced in the first pot experiment with an organic substrate compared to the other experiments. This reduction can also be explained by competition in the substrate with early colonizing saprotrophs, or by choosing a saprotrophic trophic strategy of endophytic strains caused by the availability of organic matter in the substrate.

*Fusarium* spp. had a devastating effect during germination and seedling stage of plant development, but the presence of the pathogen in the tissues of asymptomatic young plants could remain unmanifested [58,59], which is a weak point of the methodology used to evaluate plant infestation. Although molecular or biochemical analysis of the samples would be required for the presence of the pathogen, visual evaluation appears to be a suitable choice in these more than month-long experiments [59], because during that time, some of the symptoms of the infection will show. On the opposite, the browning of bases does not have to be caused directly by the *Fusarium* hyphae but as the plant response to its presence in the roots [55], but other factors and pathogens can cause this symptom [50,60]. Typical representatives of crown rot are pathogenic strains of the genus *Rhizoctonia* sp. or *Ceratobasidium* sp. [50,60,61] and predominantly in field conditions, it is difficult to determine the pathogen only by visual observation. The rate of infestation could be affected by airborne transmission caused by the increased availability of infectious particles forming on infested plants in a pot. The infected plants could serve as another source of inoculum because the major reservoirs of *Fusarium* spp. are crop residues on the substrate surface [57]. The infection process by *F. culmorum* is influenced by temperature, humidity, carbon and nitrogen availability and the ability to produce mycotoxins that may confer higher pathogenicity by inhibiting the plant defence response [28]. To understand the complexity of biological plant protection, it would be appropriate to study the impact of pathogens on plants, during their development.

*P. teres* is a serious pathogen of cereals causing the net blotch of barley negatively influencing yield and quality of grains leading to significant economic loses [62,63]. In our region, it is usually not necessary to perform artificial inoculations of *P. teres*, because the natural incidence is mostly high [64]. Conditions of the experimental year nevertheless caused the low occurrence of this pathogen. Similarly, another common pathogen *Cochliobolus sativus* [65] did not occur in our plots during the experiment. The evaluation of the presence and impact of pathogens on plants in the field is influenced, among other by factors as sowing procedures and the type of the preceding crop which, as a non-target host, can prevent the mass spread of infectious particles in the habitat by limiting the life cycle of the species-specific pathogen [60]. Although there are many influences and stressors in field conditions, oilseed rape as preceding crop played a crucial role in the results we achieved. Nonsignificant results in yield could be also caused by an excess of mineral nutrients, which affected the spreading of endophytes in the roots, as it is approved for arbuscular mycorrhiza [66]. Similar to yields, grain quality parameters could differ if colonization was higher or in stressful conditions, where the endophytic potential would play a more important role [67,68].

Application of biocontrol agents into the field is a key component of experiments [12,14,48]. Due to the results of root colonization, the spreading of the mycelia overgrown substrate seems to be an acceptable way of application for *Rh*-like fungi; however, it could be optimized to achieve colonization values comparable to pot experiments.

## 4. Materials and Methods

### 4.1. Identification of Isolates and Inoculum Preparation

Endophytic fungi were isolated from Mediterranean orchids as their mycorrhizal partners from the collection at the Masaryk University. *M. bolleyi* and *C. cereale* were isolated from wheat roots and *S. indica* was obtained from CBS culture collection (CBS 125645), Netherlands (Table 6). Inocula of tested endophytic and pathogenic fungi used in field and pot experiments were cultivated on autoclaved wheat grains for 1 month at 20 °C in the dark. For in vitro competition tests mycelia grown on Petri dishes (9 cm diameter) containing potato dextrose agar with ampicillin (1 mg/L; Carl Roth GmbH + Co. KG, Germany). Fungi were identified by sequencing the ITS regions of the rDNA. Fungal mycelia were harvested from Petri dishes, ground to a fine powder in a cooled mortar using liquid nitrogen, homogenized, and total genomic DNA was extracted using the DNeasy Plant Mini Kit (Qiagen, Hilden, Germany). The concentration of DNA was measured with Qubit (Thermo Fisher Scientific, Waltham, USA) and DNA was diluted to concentration 10 ng μL^−1^. PCR reactions were carried out in 20 mL volumes containing 10 ng of fungal DNA. The reaction mixture consisted of 0.2 mM of dNTP, 0.2 mM, 1 U of Taq polymerase (Thermo Fisher Scientific, Waltham, USA) and each of ITS1/ITS4 primers [69]. Reaction buffer consisted of 75 mM Tris-HCl, 20 mM (NH_4_)_2_SO_4_ and 2.5 mM MgCl_2_ (Thermo Fisher Scientific, Waltham, USA). PCR was conducted under the following cycling conditions: initial denaturation at 94 °C for 5 min, 35 cycles of denaturation (94 °C, 1 min), annealing (57 °C, 1 min) and the final extension in 72 °C for 5 min. PCR products (10 μL) were analysed using agarose gel electrophoresis. The amplification products were sequenced by Biocev, Czech Republic. Analysed sequences were deposited in GenBank and their accession numbers are listed in Table 6. The sequence comparison with sequences from GenBank identified isolates with 91.95–100.00% identity.

The pathogenic fungi used in the experiments are part of the collections of the Agrotest fyto, Ltd. *F. culmorum* tribe KM16902, described by Antalová et al. [39] was used in in vitro competition tests and pot experiments with wheat. The isolate of *P. teres* tribe Ptt17, analyzed by Matušinský et al. [70] was used only in in vitro experiment as a slowly growing pathogen for comparison with fast growing *F. culmorum*. For in vitro tests, the mycelia were grown on Petri dishes. The inoculum used in the first and second pot experiments was formed by grain infected with *F. culmorum* collected from plants that had been sprayed during mid-anthesis phase by *F. culmorum* macroconidia at concentration 5 × 10^5^ conidia mL^−1^. In the third pot experiment, an inoculation dose was prepared by shaking the *F. culmorum* overgrown grain in water and diluted to the concentration 5 × 10^5^ conidia mL^−1^.

### 4.2. Competitive In Vitro Test

Petri dishes (9 cm diameter) containing potato dextrose agar with ampicillin were used for the experiment. The fragments of endophytic isolates were placed on Petri dishes earlier than pathogens due to slower growth and acclimation 1 cm from the edge of the dish. After one week, the inoculum of either *F. culmorum* or *P. teres* was added on the opposite side 1 cm from the edge, i.e., the distance between fragments was 6 cm. Then, the fungi were co-cultivated for one week and evaluated visually for the presence of an inhibition zone between the isolates tested and *F. culmorum* or for the colour change of *P. teres* mycelia. There were seven treatments, i.e., five treatments with different endophytic fungi and two treatments with either *M. bolleyi* or *C. cereale* (Table 6) in six replications.

### 4.3. Pot Experiments in a Growth Chamber

All pot experiments were done using wheat (*Triticum aestivum*) variety Tobak, which is sensitive to the infectious pressure of *Fusarium* species. The experiments were performed in polyethylene pots (14 × 18 × 6 cm), 100 grains were sown in each pot. In experiment 1 and 2, we used grain infected with *F. culmorum* collected from plants that had been sprayed during mid-anthesis phase by *F. culmorum* macroconidia at concentration 5 × 10^5^ conidia mL^−1^. In experiment 3, uninfected grains were used. For the fungicidal treatment, a fungicide effective against *Fusarium* species was used, i.e., Kinto Duo (BASF SE) containing two active substances, prochloraz 55.1 g/L (group: imidazoles) and triticonazole 20.0 g/L (group: triazoles), at the dose of 2 mL/kg of wheat grains. The evaluation of the pot experiment took place between phases BBCH 12–14 regardless of their duration.

#### 4.3.1. Experiment 1

The first experiment had been running for 6 weeks. Infected wheat grains were sown into pots filled with a mixture of clinoptilolite (natural zeolite, particle size 1–2.5 mm; Zeocem, Bystré, Slovakia) and universal garden substrate (Rašelina, a.s., Soběslav, Czech Republic) in 1:1 (*v/v*) ratio. Inocula of endophytic fungi (mycelia covered grains) were added into the substrate before the wheat sowing, 15 g per pot and mixed with the substrate. The pots were put into plastic bags to maintain moisture and placed randomly in a translucent climatic box with temperature fluctuating between 12 and 15 °C. After 2 weeks, plastic bags were removed and plants were cultivated for another four weeks, re-randomizing the position of pots once a week, watered by 50 mL tap water every week and the temperature was elevated to 15–17 °C. There were seven treatments, i.e., one chemically or biologically untreated control, five inoculated with different endophytic fungi (Table 6), and one with wheat grains treated with the fungicide Kinto Duo. The evaluated parameters were the number of plants emerged and the number of vital plants without necrotic and chlorotic changes in shoots, regardless of the symptoms occurring on the stalk bases (Figure 2). There were three replicates per treatment.

#### 4.3.2. Experiment 2

The second experiment had been running for 4 weeks. Infected wheat grains were sown into pots filled with a mixture of clinoptilolite (natural zeolite, particle size 1–2.5 mm; Zeocem, Bystré, Slovakia) and Perlite—an amorphous volcanic glass (AGRO CS a.s., Říkov, Czech Republic) in 1:1 (*v/v*) ratio. The mycelium covered grains were shaken in water and the mycelia detached from the grains was filtered through and served as a source of inoculum. A total of 15 g of mycelia per pot were trapped on the sieve and mixed with the substrate. The pots were watered with 100 mL of tap water and placed into a chamber with controlled temperature. During the first week, the plants were kept in the dark at 10 °C, after that, the photoperiod was set on 14/10 light/dark regime, using fluorescent lamps as a light source, and the temperature was elevated to 12 °C. In the third week, the temperature was elevated to 15–17 °C and in the last week to 17–19 °C. Re-randomization has been done once a week and plants were watered by 100 mL of tap water weekly. There were nine treatments, i.e., one chemically or biologically untreated control, five inoculated with different endophytic fungi, two treatments inoculated with either *M. bolleyi* or *C. cereale* (Table 6)*,* and one with wheat grains treated with the fungicide Kinto Duo. The evaluated parameters were the number of symptomless plants, the number of vital plants without necrotic and chlorotic changes in shoots, regardless of the symptoms occurring on the stalk bases (Figure 2) and dry shoot mass per plant (mg). There were three replicates per treatment.

#### 4.3.3. Experiment 3

The third experiment had been running for 6 weeks. Surface sterilized (5 min in 1% NaClO) wheat grains, were sown into pots and cultivated under the same conditions as in the second experiment as regard substrate composition, temperature, photoperiod regime and watering. The temperature had risen to 17–19 °C in the fourth week and was kept until the end of the experiment. After 10 days of cultivation, plants in all treatments except one (untreated control) were rinsed with *F. culmorum* conidia, a total of 1 × 10^7^ conidia per pot. There were 11 treatments: control with *F. culmorum* application, untreated control without *F. culmorum* application, five inoculated with different endophytic fungi, three inoculated with *M. bolleyi*, *C. cereale* or *S. indica* (Table 6)*,* and one treated with the fungicide Kinto Duo. The evaluated parameters were the number of plants emerged, the number of vital plants without necrotic and chlorotic changes in shoots, regardless of the symptoms occurring on the stalk bases (Figure 2), total dry shoot mass in pots (g) and dry shoot mass per plant (mg). There were three replicates per treatment.

#### 4.3.4. Evaluation

The evaluation was done by counting the number of seedlings that were divided into distinct groups depending on crown rot expression (Figure 2) and measuring a dry mass of plants in one pot or counted to a mean plant mass. The modified methodology of the degree of plants infestation by *F. culmorum* according to Beccari et al. [59] was used. Categories 1 and 2 include necrotic and chlorotic germinated plants. Category 3 covers plants with symptoms like the browning of the root neck that indicates the pathogen invasion. In category 4, there are plants with a little brown spot on the top of the root neck. Category 5 indicates plants with no visual symptoms. These 5 categories were coupled into 3 groups—category 5 as symptomless plants, 3–5 categories as vital plants, categories 1–5 as all plants emerged.

**Figure 3:**
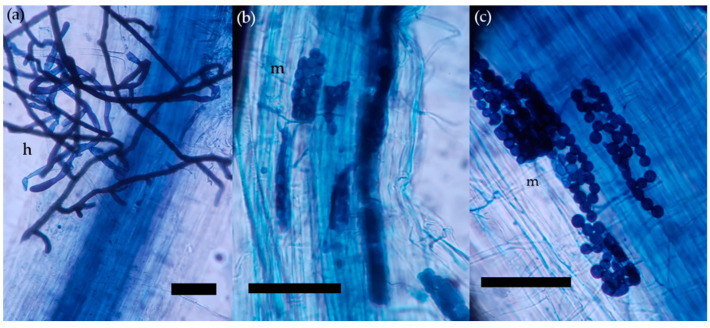
Endophytic fungi in wheat roots one month after inoculation. *Ceratobasidium* sp. 2015/1 (**a**) mostly grows extra-radically, only with few hyphae penetrating the epidermis. *Tulasnella* sp. 2016/7 (**b**) and *Tulasnella* 2016/11 (**c**) form monilioid cells inside plant cortical or epidermal tissue. Stained with trypan blue in lactoglycerol. The bars represent 100 µm. m—monilioid cells, h—hyphae.

Samples of roots were taken to evaluate the colonization by endophytic fungi (Figure 3). After harvesting, the roots were transferred into 70% ethanol to be fixed. Colonization of roots by the endophytic fungus was assessed by microscopic examination (200× magnifications) of roots cleared in 2.5% KOH, acidified in 1% HCl and stained with 0.05% trypan blue in lactoglycerol [71]. The results are expressed as a percentage of the root’s length colonized by an endophytic fungus.

### 4.4. Field Experiment

The experiment began in the first half of October 2019 with the sowing of winter barley (Fabian variety) in Kroměříž after oilseed rape as a preceding crop. Before sowing, endophytic fungi inoculation doses consisting of mycelium-covered sterilized wheat grains and another mycelium-covered organic substrate (wheat and corn biomass) was applied to a 10 square meter experimental field in the dosage of 150 g of inoculum per plot. The dosage of grains fungicide treatment was the same as in pot experiments. No other application of pesticides, except growth regulator treatment to prevent lodging of stems, was applied. There were eight treatments: untreated control, five inoculated by endophytic fungi, one inoculated with *S. indica* (Table 6) and one with barley grains treated with the fungicide Kinto Duo. There were four replicates per treatment. The evaluated parameters were the fresh and the dry weight of plants harvested at the stage of milky ripeness of the grain in early June 2020, when the plants were fully green (BBCH 73–75). It was taken 12 plants per treatment to determine the fresh mass and the dry mass of shoots. The natural occurrence of pathogens during the season by observation and the percentage of root colonization by the endophytes were also evaluated. The harvest took place in the second week of July 2020. The evaluation was done by converting the actual harvest weight and moisture to a relative weight in 15% moisture volume, then by evaluating the bulk density and the weight of a thousand grains.

### 4.5. Statistical Analysis

Data were analysed using STATISTICA 12 (TIBCO Software Inc., Palo Alto, USA). The effects of inoculation with endophytes or seed treatment with Kinto Duo on a total number of plants, their health status and the dry mass weight was assessed by one-way analysis of variance (ANOVA). All experiments were evaluated separately due to different cultivation conditions. Prior to ANOVA, the normality of the residuals was tested using the Shapiro-Wilk test and the homogeneity of variances was tested by combined Bartlett’s, Cochran’s, and Hartley’s tests. To meet these assumptions, the root colonization in the field experiment was log-transformed and the dry mass per plant in the third pot experiment was square-root transformed before ANOVA. The differences among means were assessed using *LSD*_0.05_ test. Data are presented in the form mean ± standard deviation (*n* = number of replicates) thorough the whole text.

## 5. Conclusions

In this work, we studied the potential of *Rh*-like fungi to act against cereal pathogens, mainly against *F. culmorum*. We revealed that the results obtained in vitro—the antagonistic effect of *M. bolleyi* or *C. cereale* against *F. culmorum,* do not correspond to the results of the pot experiments, in which *M. bolleyi* did not affect and *C. cereale* reduced the values of parameters tested. This may be due to the pathogenic nature of *C. cereale.* On the other hand, the well-known mutualistic *Rh*-like fungus *S. indica* substantially reduced the wheat damage. Compared to the fungi mentioned above, the isolates newly obtained from orchid mycorrhizas were intermediate as regard their protective effects against damage caused by *F. culmorum*. Our results in pot experiments indicate the potential for use in biological plant protection if an endophytic association is formed before a pathogen occurs. Successful root colonization by endophytic *Rh*-like fungi in pot experiments using both infected and uninfected grains and their different effect on plants point to the hidden factors of this association and it is necessary to observe the response of individual plants, especially at the biochemical level. Our work demonstrates clearly that the evaluation of the potential of *Rh*-like fungi under field conditions needs extensive experimentation as there are many factors which affect the results (relatively low natural infective pressure in our case). However, considering the results from pot experiments, simple production of inocula of these fungi (due to their saprotrophic capabilities) and successful colonization of wheat and barley provide *Rh*-like fungi that promise biological agents suitable for the next set of extensive testing.

## Figures and Tables

**Table 1:** Evaluation of in vitro tests of fungal isolates against plant pathogens *Fusarium culmorum* and *Pyrenophora teres*. +: presence, -: absence. The parameters of inhibition zone between fungal isolates and *F. culmorum*, reduction of *Fusarium* growth and ability to cause degradation of *P. teres* mycelium were evaluated.

Treatment	Inhibition Zone with *Fusarium culmorum*	Reduction of *Fusarium culmorum* Growth	Causing Degradation of *Pyrenophora teres*
*Ceratobasidium* 2015/1	-	-	+
*Tulasnella* 2015/2	-	-	-
*Tulasnella* 2016/2	-	-	-
*Tulasnella* 2016/7	-	-	-
*Tulasnella* 2016/11	-	-	+
*Microdochium bolleyi*	+	-	-
*Ceratobasidium cereale*	-	+	+

**Table 2:** Significant evaluated parameters of Experiment 1 include numbers of plants emerged from 100 grains contaminated with *Fusarium culmorum* (tribe KM16902) sown, and numbers of vital plants lacking any symptoms of necrosis or chlorosis caused by *F. culmorum*. Data represent means ± *SD* (*n* = 3) followed by the same letter (within the column) if there was no statistical difference according to *LSD*_0.05_ test.

Treatment	Number of Plants	Number of Vital Plants
*Ceratobasidium* 2015/1	45.7 ± 2.3 ^b^	24.0 ± 7.0 ^bc^
*Tulasnella* 2015/2	43.7 ± 4.9 ^b^	27.7 ± 7.2 ^b^
*Tulasnella* 2016/2	40.3 ± 5.5 ^bc^	26.0 ± 2.6 ^bc^
*Tulasnella* 2016/7	41.7 ± 2.1 ^b^	32.3 ± 5.1 ^b^
*Tulasnella* 2016/11	32.0 ± 6.1 ^c^	17.0 ± 4.4 ^c^
Kinto Duo	89.3 ± 2.1 ^a^	89.0 ± 2.0 ^a^
Control (with *Fusarium*)	47.7 ± 10.1 ^b^	29.0 ± 7.9 ^b^

**Table 3:** Percentage of wheat root colonization in all pot experiments and barley root colonization in field conditions, after inoculation with varia fungi isolates (Figure 3). Data are expressed as means ± *SD* (*n* = 3) followed by the same letter (within the column) if there was no statistical difference according to *LSD*_0.05_ test. ND—not detected.

Treatment	Experiment 1	Experiment 2	Experiment 3	Field
*Ceratobasidium* 2015/1	3.3 ± 2.9 ^b^	13.3 ± 5.5 ^de^	25.7 ± 10.3 ^c^	19.3 ± 6.7 ^cd^
*Tulasnella* 2015/2	3.3 ± 2.9 ^b^	30.0 ± 11.5 ^bc^	79.7 ± 12.9 ^a^	5.7 ± 1.5 ^ab^
*Tulasnella* 2016/2	12.3 ± 4.9 ^a^	41.0 ± 7.0 ^b^	74.0 ± 8.0 ^a^	16.7 ± 10.1 ^cd^
*Tulasnella* 2016/7	8.3 ± 3.5 ^ab^	81.3 ± 6.7 ^a^	87.0 ± 3.6 ^a^	2.7 ± 1.2 ^a^
*Tulasnella* 2016/11	12.0 ± 4.6 ^a^	83.3 ± 4.2 ^a^	87.7 ± 3.8 ^a^	29.3 ± 16.4 ^d^
*Ceratobasidium cereale*		5.3 ± 2.5 ^e^	55.7 ± 7.1 ^b^	
*Microdochium bolleyi*		27.7 ± 12.9 ^c^	75.0 ± 9.8 ^a^	
*Serendipita indica*			55.0 ± 6.6 ^b^	11.0 ± 2.6 ^bc^
Kinto Duo	ND	ND	ND	ND
Control (with *Fusarium*)	4.3 ± 4.0 ^b^	18.7 ± 3.5 ^cd^	51.0 ± 14.0 ^b^	
Control (without *Fusarium*)			ND	ND

**Table 4:** Significant evaluated parameters of Experiment 2 include number of symptomless (no observable impact of *Fusarium culmorum*) plants emerged from 100 grains contaminated with *F. culmorum* (tribe KM16902) sown, number of vital plants (lacking any symptoms of necrosis or chlorosis caused by *F. culmorum*), and dry shoot mass per emerged plant (mg). Data represent means ± *SD* (*n* = 3) followed by the same letter (within the column) if there was no statistical difference according to *LSD*_0.05_ test.

Treatment	Number of Symptomless Plants	Number of Vital Plants	Dry Shoot Mass Per Plant (mg)
*Ceratobasidium* 2015/1	16.0 ± 5.3 ^ab^	64.3 ± 12.9 ^b^	16.4 ± 0.5 ^ab^
*Tulasnella* 2015/2	5.7 ± 6.0 ^c^	54.0 ± 10.0 ^b^	16.3 ± 0.7 ^ab^
*Tulasnella* 2016/2	12.3 ± 11.0 ^bc^	60.7 ± 15.5 ^b^	17.4 ± 1.1 ^a^
*Tulasnella* 2016/7	7.3 ± 6.1 ^bc^	54.0 ± 10.5 ^b^	15.8 ± 0.6 ^bc^
*Tulasnella* 2016/11	8.7 ± 5.5 ^bc^	60.7 ± 14.0 ^b^	15.9 ± 1.6 ^abc^
*Ceratobasidium cereale*	0.0 ± 0.0 ^d^	68.0 ± 1.0 ^b^	14.6 ± 0.9 ^cd^
*Microdochium bolleyi*	20.0 ± 5.3 ^ab^	70.0 ± 13.0 ^b^	16.1 ± 0.1 ^abc^
Kinto Duo	47.3 ± 22.7 ^a^	88.7 ± 3.1 ^a^	14.1 ± 0.7 ^d^
Control (with *Fusarium*)	17.0 ± 5.6 ^ab^	58.3 ± 5.1 ^b^	17.0 ± 1.2 ^ab^

**Table 5:** Evaluated parameters of Experiment 3 include numbers of plants emerged from 100 grains sown, and numbers of vital plants lacking any symptoms of necrosis or chlorosis caused by *Fusarium culmorum*. Dry shoot mass (g) and dry shoot mass per emerged plant (mg). Data represent means ± *SD* (*n* = 3) followed by the same letter (within the column) if there was no statistical difference according to *LSD*_0.05_ test.

Treatment	Number of Plants	Number of Vital Plants	Dry Shoot Mass (g)	Dry Mass Per Plant (mg)
*Ceratobasidium* 2015/1	73.3 ± 16.1 ^e^	51.0 ± 12.3 ^e^	0.922 ± 0.22 ^cd^	13.3 ± 5.7 ^abc^
*Tulasnella* 2015/2	89.0 ± 7.0 ^abcd^	56.0 ± 9.8 ^e^	1.188 ± 0.113 ^b^	13.4 ± 1.0 ^abc^
*Tulasnella* 2016/2	92.0 ± 4.6 ^abc^	81.0 ± 8.5 ^bc^	1.198 ± 0.104 ^b^	12.7 ± 1.4 ^bc^
*Tulasnella* 2016/7	75.7 ± 8.4 ^de^	60.3 ± 8.5 ^de^	0.955 ± 0.109 ^cd^	13.0 ± 0.8 ^bc^
*Tulasnella* 2016/11	93.3 ± 1.5 ^abc^	75.3 ± 9.0 ^cd^	1.181 ± 0.121 ^b^	12.7 ± 1.5 ^bc^
*Ceratobasidium cereale*	56.7 ± 3.1 ^f^	5.0 ± 3.6 ^g^	0.428 ± 0.141 ^e^	7.5 ± 1.5 ^d^
*Microdochium bolleyi*	85.3 ± 9.3 ^bcde^	60.7 ± 8.1 ^de^	0.998 ± 0.121 ^bcd^	11.6 ± 0.8 ^bc^
*Serendipita indica*	99.3 ± 0.6 ^a^	97.3 ± 2.3 ^a^	1.512 ± 0.067 ^a^	15.2 ± 0.6 ^ab^
Kinto Duo	90.7 ± 8.7 ^abc^	94.7 ± 4.0 ^ab^	1.108 ± 0.032 ^bc^	11.7 ± 1.4 ^bc^
Control (with *Fusarium*)	81.3 ± 10.0 ^cde^	30.3 ± 16.8 ^f^	0.831 ± 0.177 ^d^	10.2 ± 2.1 ^cd^
Control (without *Fusarium*)	95.3 ± 3.8 ^ab^	89.3 ± 8.1 ^abc^	1.504 ± 0.089 ^a^	16.6 ± 1.1 ^a^

**Table 6:** The origin of endophytic isolates used as individual treatments, internal code, species according to the internal transcribed spacer and accession number in the genetic sequence database.

Isolate Origin	Internal Code	Species	Accession Number GenBank
*Ophrys bombyliflora*	2015/1	*Ceratobasidium* sp.	MW488152
*Orchis italica*	2015/2	*Tulasnella* sp.	MW485781
×*Serapicamptis capitata*	2016/2	*Tulasnella* sp.	MW485782
*Serapias lingua*	2016/7	*Tulasnella* sp.	MW485784
*Serapias lingua*	2016/11	*Tulasnella* sp.	MW485827
*Triticum aestivum*	18–301	*Microdochium bolleyi*	MW485763
*Triticum aestivum*	18–300	*Ceretobasidium cereale*	MW485776
*Prosopis juliflora* rhizosphere [11]	CBS 125645	*Serendipita indica*	MH863568

## Data Availability

All data are contained within the article.
